# Diverse Clinical Manifestations and Challenges of Mucormycosis: Insights From Serial Cases

**DOI:** 10.1093/ofid/ofad527

**Published:** 2023-10-24

**Authors:** Marisa C Nielsen, Filipe M Cerqueira, Sri Bharathi Kavuri, Caitlin M Raymond, Aeman Muneeb, Andrzej S Kudlicki, Shafaq Tariq, Mingru Liu, Andrew L Routh, Suimin Qiu, Ping Ren

**Affiliations:** Department of Pathology, University of Texas Medical Branch, Galveston, Texas, USA; Department of Pathology and Laboratory Medicine, Boston Medical Center and Boston University Chobanian & Avedisian School of Medicine, Boston, Massachusetts, USA; Department of Pathology, University of Texas Medical Branch, Galveston, Texas, USA; Department of Pathology, University of Texas Medical Branch, Galveston, Texas, USA; Department of Pathology, University of Texas Medical Branch, Galveston, Texas, USA; Department of Radiology, University of Texas Medical Branch, Galveston, Texas, USA; Department of Biochemistry and Molecular Biology, University of Texas Medical Branch, Galveston, Texas, USA; Department of Internal Medicine-Infectious Diseases, University of Texas Medical Branch, Galveston, Texas, USA; Department of Pathology, University of Texas Medical Branch, Galveston, Texas, USA; Department of Biochemistry and Molecular Biology, University of Texas Medical Branch, Galveston, Texas, USA; Department of Pathology, University of Texas Medical Branch, Galveston, Texas, USA; Department of Pathology, University of Texas Medical Branch, Galveston, Texas, USA

**Keywords:** *Apophysomyces*, clinical mycology, fungal infections, mucormycetes, mucormycosis

## Abstract

Mucormycosis is a severe and potentially life-threatening infection caused by a group of fungi classified as mucormycetes within the scientific order Mucorales. These infections are characterized by rapid and invasive fungal growth, presenting significant treatment challenges. Here we present 5 cases encountered from 2018 to 2022 at the University of Texas Medical Branch in Galveston, Texas, including a novel *Apophysomyces* species. These cases illustrate the diverse clinical manifestations of mucormycosis, including pulmonary, rhino-cerebral, gastrointestinal, and soft tissue involvement. Our investigation incorporates information provided by a multidisciplinary team of clinical collaborators, emphasizing the findings from radiology, histopathology, and microbiology. Given the escalating global incidence of mucormycosis, it is crucial for clinicians to become familiar with associated clinical findings, comorbidities, and risk factors to facilitate prompt recognition, appropriate diagnostic testing, and timely initiation of treatment.

Mucormycosis, an aggressive and potentially life-threatening fungal infection, is caused by fungi within the Mucorales order. While historically considered rare, these infections present significant clinical challenges. They are primarily opportunistic and commonly associated with immunosuppression, particularly in individuals with uncontrolled diabetes [1–4]. However, documented cases have also been observed in otherwise healthy individuals [4]. Notable genera responsible for mucormycosis include *Rhizopus, Mucor, Rhizomucor, Apophysomyces, Cunninghamella*, *Syncephalastrum*, *Lichtheimia,* and *Saksenaea* [1].

Mucormycosis exhibits diverse manifestations, including pulmonary, rhino-orbital-cerebral, gastrointestinal, and soft tissue infections, all of which can progress to disseminated and potentially fatal infections [5]. Pulmonary mucormycosis is commonly observed in patients with neutropenia, solid organ transplants, and stem cell transplants [2, 5, 6]. Rhino-orbital and rhino-cerebral infections are frequently associated with uncontrolled diabetes or kidney transplants [6, 7]. Gastrointestinal mucormycosis is more prevalent among premature infants and less commonly reported in adults [8, 9]. Soft tissue infections typically result from direct inoculation of fungal propagules into the skin through traumatic injuries involving soil-contaminated debris/equipment, surgical sites, or burn wounds [1]. Invasive mucormycosis is characterized by fungal invasion of the blood vessels visualized on histopathology stains, leading to thrombosis and tissue necrosis [10]. Accurate identification at the species level is crucial and should be performed using phenotypic, biochemical, and/or molecular testing methods.

The overall mortality rate of mucormycosis is estimated to be ∼45%, although it varies significantly depending on the site of infection: disseminated (97%), gastrointestinal (85%), pulmonary (76%), rhino-cerebral (62%), cutaneous (31%) [1, 11]. Prompt treatment plays a crucial role in patient survival and typically involves surgical debridement along with antifungal therapy [1]. Amphotericin B is the preferred antifungal; however, certain species, such as *Apophysomyces* spp., *Rhizopus* spp., and *Cunninghamella* spp., may exhibit high minimum inhibitory concentrations (MICs), posing significant treatment challenges [12].

This study presents 1 representative case each of pulmonary, rhino-cerebral, gastrointestinal, and soft tissue mucormycosis plus a newly identified *Apophysomyces* species encountered at the University of Texas Medical Branch (UTMB) in Galveston, Texas, between 2018 and 2022.

## METHODS

### Phenotypic Characterization

Fungal isolates were cultured on Potato Dextrose Agar (PDA) and incubated at 25°C. Czapek-Dox agar (Sigma, St. Louis, MO, USA) was used to induce sporulation for the isolate obtained from case 2. Microscopic examination of cultures was performed on slide cultures stained with lactophenol cotton blue (LPCB).

### Fungal DNA Extraction and Polymerase Chain Reaction

DNA extraction was performed using PrepMan Ultra Sample Preparation Reagent (ThermoFisher Scientific, Waltham, MA, USA) following the manufacturer's instructions. Briefly, fungal mycelia were combined with MP Ceramic Sphere (MP Biomedicals, Santa Ana, CA, USA) and PrepMan Ultra Sample Preparation Reagent. The mixture was homogenized using the FastPrep-24 Sample Preparation Instrument (MP Biomedicals, Santa Ana, CA, USA). After heat inactivation at 95°C (10 minutes) and cooling to room temperature (2 minutes), the tube was centrifuged at the highest speed (2 minutes). The fungal DNA was stored at −20°C.

For PCR, 25-µL polymerase chain reaction (PCR) mixtures were assembled with specific primer pairs targeting the internal transcribed spacer (ITS), the D1 and D2 (D1/D2) domains of the 28S nuclear ribosomal RNA (rRNA) gene, and a fragment of the histone H3 gene (H3), respectively. After gel electrophoresis confirmation, ExoSAP-IT Express (ThermoFisher Scientific, Waltham, MA, USA) was used for PCR product purification, and sequencing was performed by ACGT, Inc. (Wheeling, IL, USA).

### Next-Generation Sequencing

Purified DNA was sheared using a Covaris g-tube to yield DNA fragments ∼8–10 k bp in length. Sheared DNA (1 μg) was prepared for sequencing on the Oxford Nanopore Technologies (ONT) platform using the SQK-LSK109 protocol. Briefly, DNA was end-prepped and A-tailed using the NEBNext Endprep Module and then directly ligated to the ONT adaptor using NEB Blunt TA ligase mix. After bead clean-up, libraries were loaded per the manufacturer's instructions, and sequencing was performed for >24 hours using MinKNOW. Raw FAST5 data were base-called in “high accuracy” mode using GPU-accelerated Guppy to yield FASTQ data.

### Phylogenetic Analysis

Phylogenetic analyses were conducted using 3 clinical isolates (cases 1, 2, and 3) along with 13 species of Mucorales for which ITS and D1/D2 sequence data are available in the National Center for Biotechnology Information (NCBI) GenBank. To establish the relationship of the novel *Apophysomyces* species identified in case 1, H3 gene sequences were concatenated with ITS and D1/D2 sequences of other known *Apophysomyces* isolates. Two phylogenetic trees were constructed using the maximum likelihood method with MEGA XI software [26].

Additional phylogenetic analysis, using NGS-based genomic information, was performed using conserved benchmark genes that could be uniquely identified within the contig-level assembly of the genome.

The provisional genome assembly of the novel *Apophysomyces* species consisted of 4838 contigs. The BUSCO 5.4.7 (Benchmarking Universal Single-Copy Orthologs) software package was used to identify candidate genomic features [27]. Running BUSCO against the mucoromycota_odb10 lineage database resulted in 405 complete and nonduplicated predicted orthologs out of 1614 total benchmark orthologs. The remaining genes were either fragmented, duplicated, or missing from the prediction. BUSCO was also used to predict conserved genes in published draft genomes of other *Apophysomyces* species not included in mucoromycota_odb10: *Apophysomyces elegans, Apophysomyces ossiformis, Apophysomyces trapeziformis, Apophysomyces variabilis,* and 3 uncharacterized genomes: *Apophysomyces* spp. BC1015, BC1021, and BC1034.

Clustal Omega for protein was used to align the protein sequences of the orthologous groups with standard parameters, and the output was processed using the FastTree algorithm (http://www.microbesonline.org/fasttree/).

## RESULTS

### Clinical Presentations

#### *Novel* Apophysomyces *Species Soft Tissue Infection (Case 1)*

**Figure 1.**
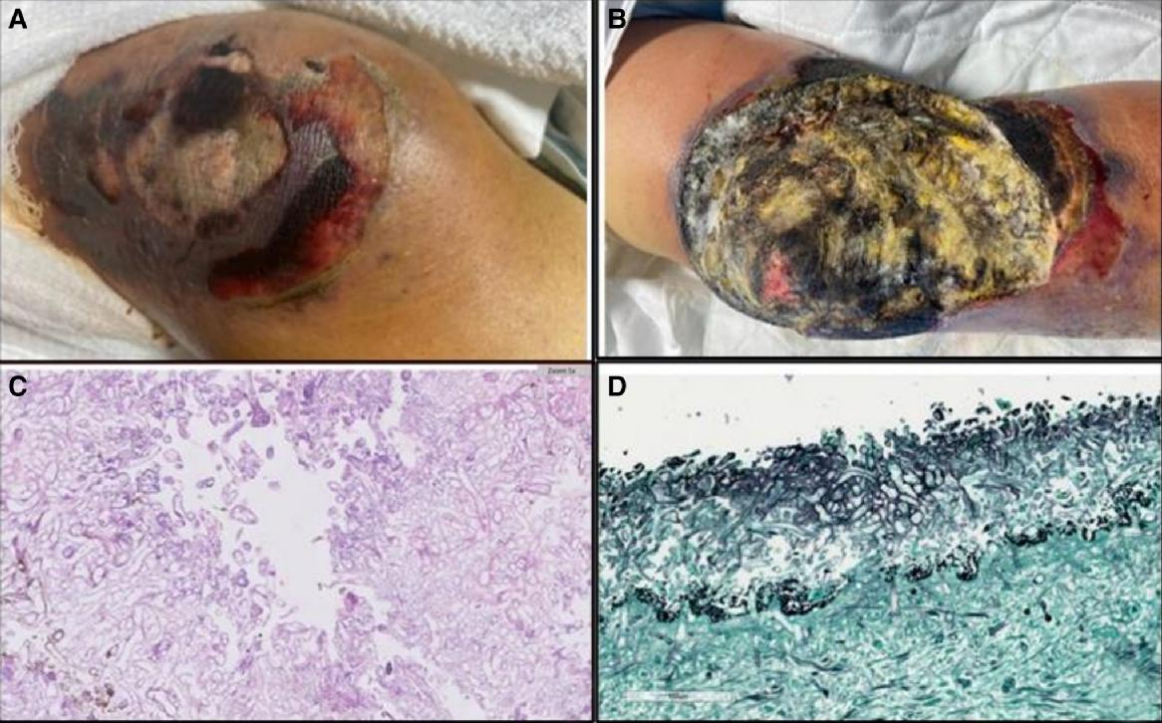
The patient's right knee (case 1) on initial presentation (*A*) and 5 days later, after 2 surgical debridements (*B*). Dense fungal hyphae seen on H&E stain 400× (*C*) and GMS 400× (*D*). Abbreviations: GMS, Grocott methenamine silver; H&E, hematoxylin and eosin.

A 59-year-old male with a medical history (MH) of chronic hepatitis C infection, liver cirrhosis, and previous right knee surgeries presented to the emergency department (ED) with knee cellulitis and necrosis following a fall in his yard 2 days prior. Initial debridement of the affected area revealed the presence of *Enterococcus faecalis* first. The patient was promptly started on vancomycin along with other antibiotics upon admission, with vancomycin being continued as the sole antibiotic after *E. faecalis* identification. Additionally, voriconazole was initiated empirically before the mucormycete was isolated from the fungal culture. However, despite treatment, the necrotic wound persisted, leading to repeat debridement on hospital day (HD) 5. Voriconazole was switched to amphotericin B after the infectious diseases consult. The surgical pathology findings from the second debridement confirmed the presence of mucormycosis (Figure 1). Unfortunately, the patient's condition deteriorated rapidly, with the development of encephalopathy and respiratory symptoms by HD 7. The patient was transferred to the intensive care unit (ICU) and died on HD 8.

#### Apophysomyces trapeziformis *Soft Tissue Infection (Case 2)*

A 42-year-old male presented with a grade 3 open distal femur fracture with degloving from a farm tractor accident. Multiple debridements and an open reduction internal fixation were performed. Empiric treatment with voriconazole, piperacillin-tazobactam, and vancomycin was initiated. The patient's postoperative condition was favorable initially. However, on HD 9, the patient exhibited worsening pain, erythema, and swelling, which raised concerns of necrotizing fasciitis (Supplementary Figure 1). Culture results from subsequent debridement grew *A. trapeziformis*. Despite multiple debridements and treatment with voriconazole, the fungal infection continued to progress rapidly. Eventually, the decision was made to perform an above-the-knee amputation. Therapy was switched to amphotericin B for 3 weeks, resulting in the resolution of the infection. Upon discharge, the patient was prescribed posaconazole for 5 days with plans to reevaluate the duration of antifungal therapy on outpatient follow-up. Eventually, the patient experienced minimal pain at the site of infection during a 2-year follow-up period.

#### Rhizopus arrhrizus (*Formerly* R. oryzae) *Rhino-Orbital-Cerebral Infection (Case 3)*

A 68-year-old female with a MH of uncontrolled type 2 diabetes mellitus (T2DM) and glaucoma presented with worsening right-sided facial cellulitis and suspected maxillary osteomyelitis demonstrated by computed tomography (CT) images (Supplementary Figure 2). Biopsy and lesion excision of hard palate ulcers of the right inferior turbinate were performed. Histopathology examination revealed the presence of broad ribbon-like hyphae and budding yeast, and microbiology cultures grew *Candida* species and *R. arrhizus* (images not shown). On HD 3, vancomycin was discontinued, and treatment was switched to ampicillin-sulbactam and amphotericin B. However, the patient experienced cardiac arrest, requiring intubation on HD 4. Following a debridement procedure on HD 5, the patient was transitioned to palliative care and died on HD 9.

#### Rhizopus microsporus *var.* oligosporus *Pulmonary Infection (Case 4)*

**Figure 2.**
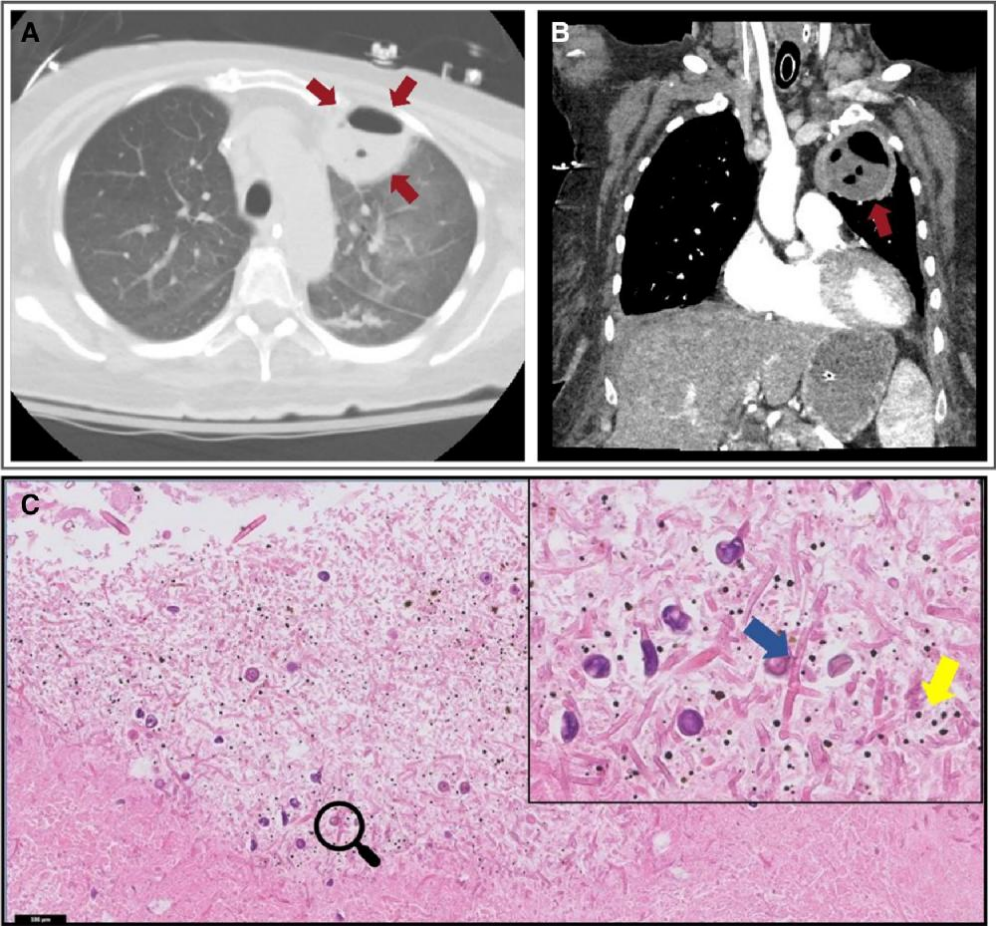
Cavitary pulmonary lesion radiology and histopathology. Axial lung (*A*) and coronal soft tissue (*B*) images from a CT angiogram of the chest demonstrate a thick-walled, peripherally enhancing cavity lesion in the anterior segment of the LUL with an air-fluid level (red arrows). *C*, H&E (200×) of the necrotic cavity in the LUL, inlay showing a higher power view of the lesion filled with inflammatory debris (yellow arrow) and fungal hyphae (blue arrow). Abbreviations: CT, computed tomography; H&E, hematoxylin and eosin; LUL, left upper lobe.

A 64-year-old female with a significant MH of T2DM, end-stage renal disease, and chronic obstructive pulmonary disease (COPD) was transferred from a long-term acute care (LTAC) facility on day 5 of mechanical ventilation due to acute hypoxic respiratory failure. The patient presented with large-volume hemoptysis caused by a mass-like infiltrate in the left upper lobe (LUL) of her lung. A chest computed tomography (CT) angiogram with intravenous contrast (Figure 2) revealed a 4.8 × 6.0 × 5.4-cm thick-walled cavitary lesion in the LUL, which was successfully managed using coil embolization. Although the initial treatment showed promise, the patient's condition deteriorated, with failed spontaneous breathing trials and episodes of pulmonary hemorrhage that eventually led to cardiac arrest. Fungal culture of a tissue biopsy obtained from the LUL lesion on HD 6 (intubation day 11) grew *Rhizopus microsporus* var. *oligosporus*. Pulmonary hygiene procedures were initiated, and the patient received amphotericin B while awaiting susceptibility results. After successful treatment, a 3-month regimen of posaconazole was initiated, resulting in complete resolution of the infection.

#### Rhizopus microsporus *Gastrointestinal Infection (Case 5)*

**Figure 3.**
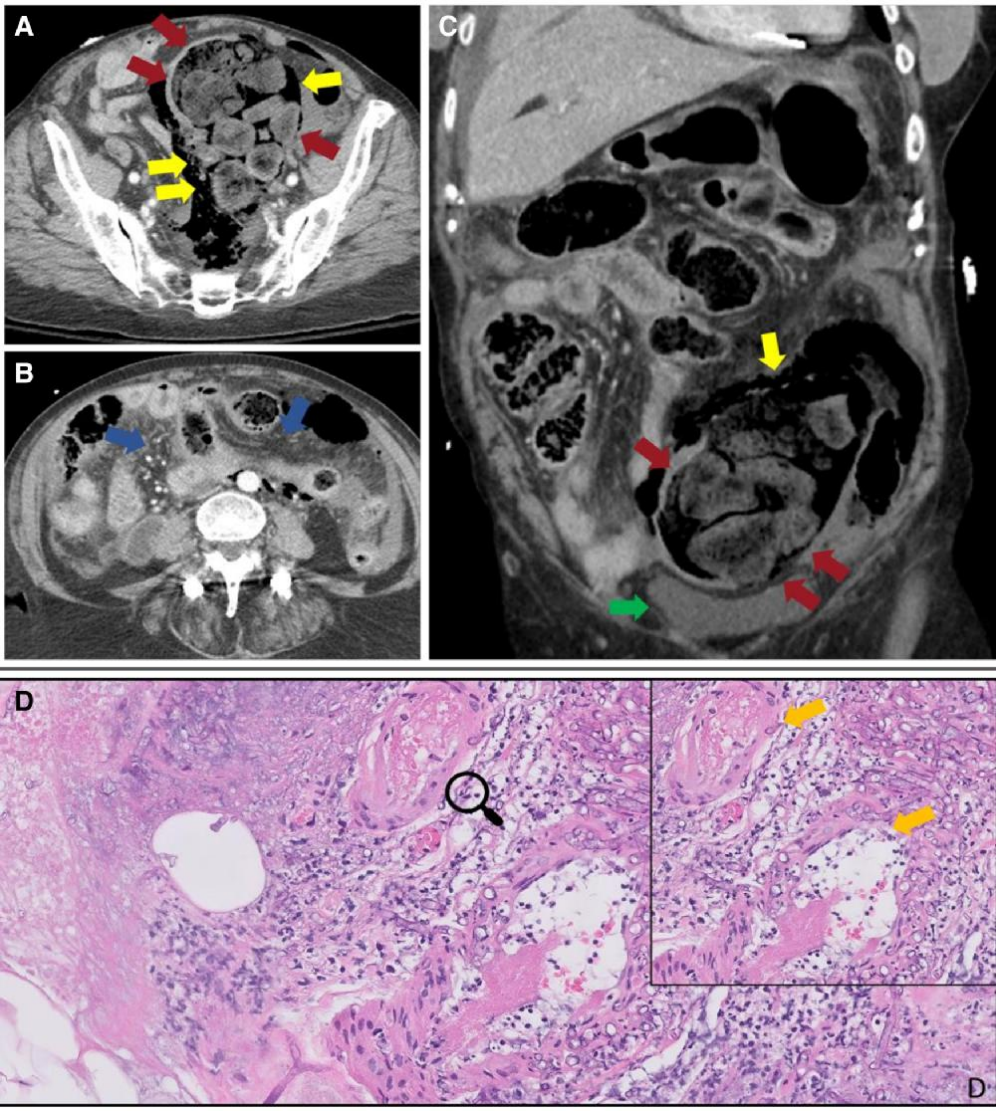
Perforated sigmoid colon with peritonitis and histopathology. Axial (*A* and *B*) and coronal (*C*) contrast-enhanced CT of the abdomen and pelvis demonstrates marked distention of the sigmoid colon with stool (red arrows). Extraluminal gas tracks along the periphery of the sigmoid colon (yellow arrow) and layering intraperitoneal free fluid (green arrow) are identified. Blue arrows indicate diffuse peritoneal fat stranding. Histopathological examination on H&E stain from the abdominal wall debridement (*D*) shows soft tissue necrosis with angioinvasive fungal hyphae (orange arrows) in a medium-sized vessel with fibrin deposits. Abbreviations: CT, computed tomography; H&E, hematoxylin and eosin.

An 81-year-old female with a MH of cardiovascular disease and adrenal insufficiency presented to the ED with 1 week of abdominal pain, constipation, nausea, and vomiting. Due to a 75% sigmoid colon perforation with stool leakage, the patient underwent sigmoidectomy and colostomy formation. However, 2 days later, the colostomy appeared dusky, and necrotic omentum was observed. A colostomy revision was performed, and the necrotic tissue was resected. Excisional biopsies of the abdominal wall and peritoneum were sent for pathological examination, revealing angioinvasive mucormycosis (Figure 3) caused by *Rhizopus microsporus,* as evidenced by the growth from abdominal fluid and abdominal wall tissue. Multiple debridements of fascial edges and skin washouts were performed, and no additional necrotic tissue was observed. However, the patchy yellow-white film in the small bowel, fascia, and omentum remained unchanged during treatment. The patient received intravenous (IV) amphotericin B for 2 weeks, followed by posaconazole. However, the patient experienced complications including delirium, atrial fibrillation with rapid ventricular response, worsening hypoxia, and ultimately succumbed to the infection on HD 19.

### Characterization of the Novel Species

**Figure 4.**
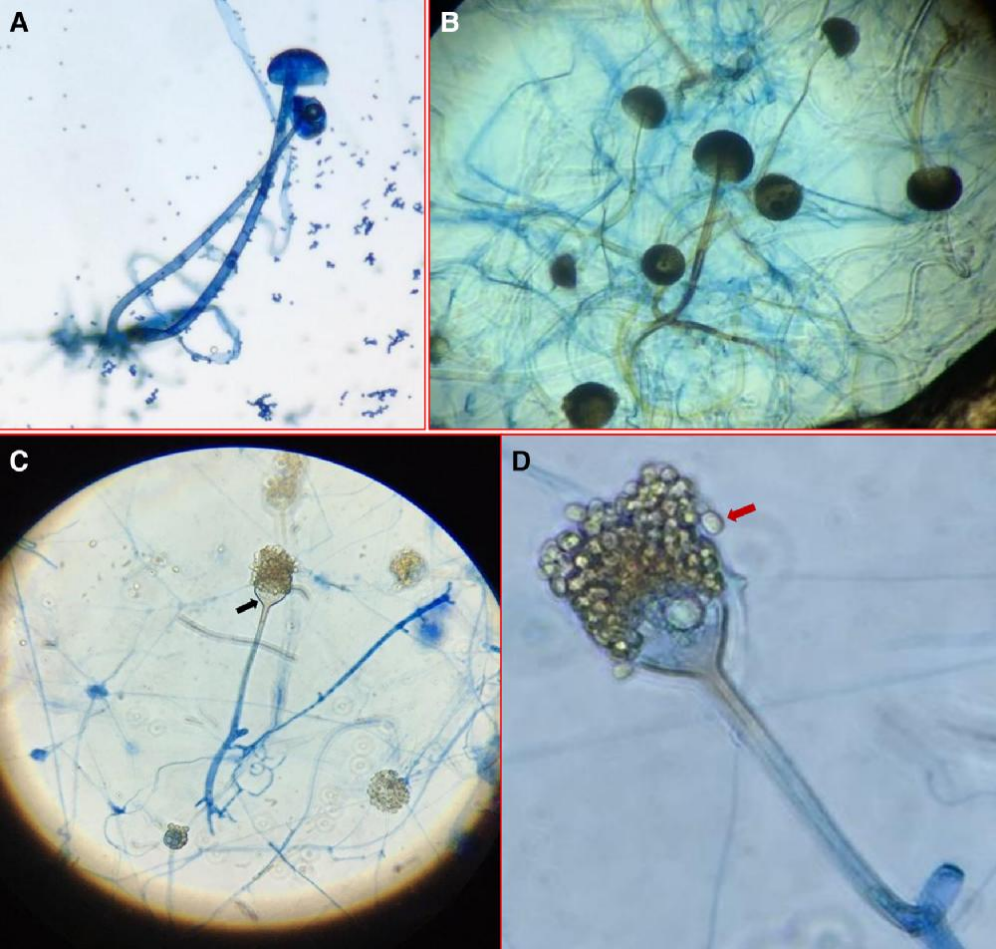
Microscopic morphology of *Rhizopus microsporus* var. *oligosporus* from case 4 (*A*, 200×), *Rhizopus arrhizus* from case 3 (*B*, 200×), and the novel *Apophysomyces* sp. from case 1 (*C*, 200×: *D*, 400×).

The microscopic morphology of *R. microsporus* var. *oligosporus* from case 4 (Figure 4*A*) and *R. ahhrizus* from case 3 (Figure 4*B*) is shown in comparison with the morphology of the novel mucormycete isolated from case 1 (Figure 4*C* and *D*). All isolates displayed primarily broad, aseptate, or pauci-septate hyphae. The novel mucormycete from case 1, stained with LPCB, exhibited sporangiophores arising from distinct foot cells. Rhizoids were located directly beneath the sporangiophores or occasionally adjacent to them (Figure 4*C*). Notable features included bell-shaped apophyses (Figure 4*C*, black arrow), slightly pigmented subapical thickening below the apophyses, and smooth-walled hyaline sporangiospores (Figure 4*D*, red arrow). The sporangiospores of the novel *Apophysomyces* species displayed a slightly trapezoidal shape when viewed from the side but appeared more cylindrical from the front, which is consistent with the characteristic morphology of this genus. In contrast, LPCB staining of *R. microsporus* var. *oligosporus* and *R. ahhrizus* exhibited round sporangia, rhizoids positioned directly beneath the sporangiophores, and no apophyses, as expected. All isolates demonstrated rapid growth, covering a substantial portion of the agar plate with white, woolly mycelia after 2 days of incubation.

Similar to other mucormycetes in the tissue, histopathological examination of the knee tissue from case 1 with the novel *Apophysomyces* sp. showed fungal elements with ribbon-like pauci-septate hyphae admixed with inflammatory cells using hematoxylin and eosin (H&E) stain (Figure 1*C*). Additionally, the fungal elements showed dark brown/black staining with Grocott methenamine silver (GMS) stain, which specifically highlights fungal structures (Figure 1*D*).

### Sequencing and Phylogenetic Analysis

The NCBI BLAST analysis of the ITS sequence (GenBank accession #OQ780364) from the fungal isolate obtained from case 1 revealed a close similarity to *A. trapeziformis* isolate HvAtITS (GenBank accession #MK841582) with 90% query coverage and 85.32% identity on 799 bases of the query sequence. The D1/D2 sequence (GenBank accession #OQ780371) of the isolate had 99% query coverage and 97.63% identity with *Apophysomyces mexicanus* CBS 136361 (GenBank accession #HG974256) on 720 bases of the query sequence. The H3 gene sequence (GenBank accession #OQ745583) of the isolate showed 100% query coverage and 99.16% identity with *A. mexicanus* CBS 136361 (GenBank accession #HG974254) on 357 bases of the query sequence.

**Figure 5.**
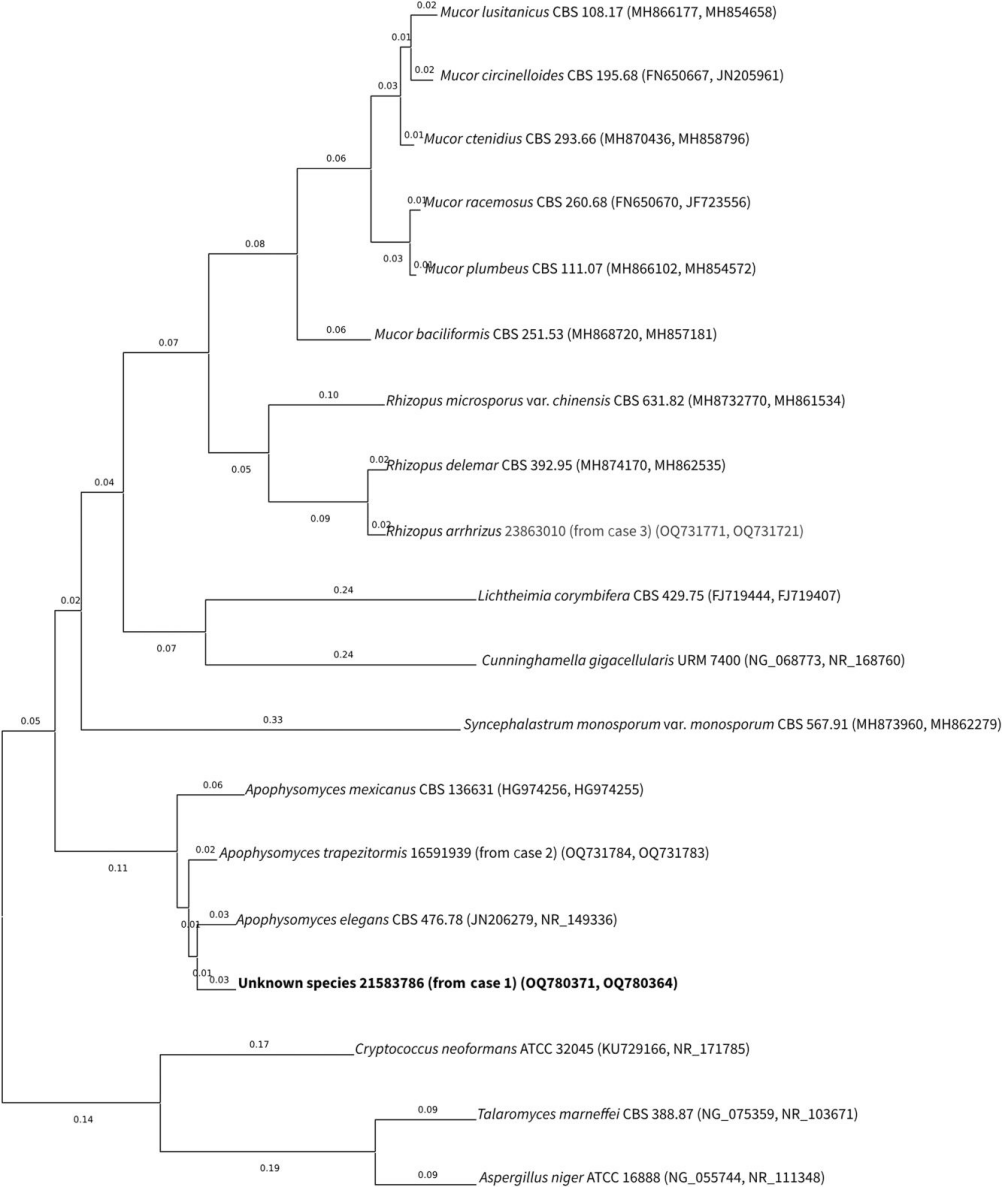
Phylogenetic tree showing the relationships of the novel species isolated from case 1 with various mucormycetes genera of *Apophysomyces, Cunninghamella, Lichtheimia, Mucor, Rhizopus,* and *Syncephalastrum*. *Apophysomyces trapeziformis* isolated from case 2 and *Rhizopus arrhizus* isolated from case 3 are included as well. Accession numbers are given as cited in the NCBI GenBank database, with the first accession numbers in the parentheses referring to the D1/D2 gene sequences and the second accession numbers in the parentheses referring to the ITS gene sequences. Abbreviation: ITS, internal transcribed spacer.

Two phylogenetic trees (Figure 5; Supplementary Figure 3) were constructed based on the availability of sequences in the NCBI GenBank database. Figure 5 represents the maximum-likelihood (ML) tree based on concatenated sequences of D1/D2 and ITS from various closely related genera of mucormycetes. It shows that the isolate obtained from case 2 and the unidentified species acquired from case 1 belong to the same clade within the *Apophysomyce*s genus. *Cryptococcus neoformans, Talaromyces marneffei,* and *Aspergillus niger* were used as outliers in this tree. Supplementary Figure 3 further demonstrated the relationship of the novel species from case 1 with other known species of *Apophysomyces,* using sequences available for D1/D2, ITS, and H3 genes. *Saksenaea vasiformis* was used as an outlier in this tree.

Phylogenetic analysis of NGS-based genomic information provided further evidence that this novel species belonged to the *Apophysomyces* genus. For most benchmark genes, the phylogeny is consistent with those derived from the ITS and D1/D2 sequences (Supplementary Figure 4).

## DISCUSSION

### Mucormycosis Cases Increase

This study highlights the discovery of a newly identified *Apophysomyces* species in a patient with multiple risk factors for mucormycosis. While mucormycosis cases have historically been rare [11], there has been a consistent increase in the number of cases over the past few decades, with a recent surge observed in individuals with coronavirus disease 2019 and those using glucocorticoids [13]. Several factors contribute to this increase, including a higher prevalence of immunosuppressed/immunocompromised individuals, an increased environmental burden of fungal spores [14], and improved diagnostic techniques and organism identification [11]. In this study, most patients had underlying comorbidities associated with mucormycosis, such as diabetes and immunosuppression. However, 1 patient without any known risk factors also developed mucormycosis, highlighting the potential for *Apophysomyces* spp. to infect immunocompetent individuals, particularly in trauma-related cutaneous infections [15]. *Saksenaea* is another mucomycete most often associated with cutaneous and subcutaneous lesions following trauma in immunocompetent hosts [16–18]. In addition, it is important to note that T2DM is a known risk factor for mucormycosis, and 2 patients in this study had T2DM. Considering the global increase in T2DM and other associated risk factors, the prevalence of mucormycosis is expected to increase [19].

### Pathogenicity

*Apophysomyces* infections, often acquired via direct cutaneous inoculation, represent a distinctive facet of mucormycosis pathophysiology. Unlike the more commonly recognized inhalation route of spore exposure, direct cutaneous inoculation involves the introduction of fungal elements through traumatic injuries, including wounds contaminated with soil or decaying vegetation. This unique mode of entry predisposes individuals to localized skin and soft tissue infections, setting *Apophysomyces* apart from other mucormycetes that frequently cause sinus and lung infections through inhalation (https://www.cdc.gov/fungal/diseases/mucormycosis/). *Apophysomyces* spp. infections, though primarily cutaneous, possess the potential for hematogenous dissemination to other anatomical sites, such as the kidney [20, 21]. Rhino-orbital-cerebral infection by *Apophysomyces* has also been reported [22, 23].

### Importance of Geographic Location and Climate Change

*Apophysomyces* species are commonly found in soil and decaying vegetation in tropical to subtropical regions with high humidity. Though mucormycosis cases caused by *Apophysomyces* spp. have a global distribution, the highest incidence rates have been reported in the Southern United States and India [24, 25]. Reportedly, India contributes to ∼60% of the global cases of *Apophysomyces* infections [5]. Our institution being located in Southeast Texas, where Mucorales are relatively common, allows for early recognition and prompt treatment of these infections. Fungi associated with disasters have the potential to spread to other geographic locations through natural forces such as wind and water. Clusters of mucormycosis cases have been reported in regions affected by major natural disasters like tornadoes, tsunamis, and volcanic eruptions [12, 26–29], even in areas where mucormycosis is not typically encountered. This presents a significant challenge for diagnosis in nonendemic areas, leading to delays in treatment and adverse patient outcomes. As the frequency of natural disasters increases due to climate change, there is likely to be a rise in mucormycosis cases among disaster survivors [30]. Additionally, many Mucorales are thermotolerant [25], and as global temperatures rise, the incidence of mucormycosis cases may also increase. Factors such as geographical residence, travel history, and environmental exposures are important considerations for diagnosis, as the clinical course, treatment, and severity of infection can vary depending on the specific causative species [11].

### Histopathology and Microbiology

Histopathology is an important tool in the diagnosis of mucormycosis as it helps assess the extent of tissue invasion [11, 31]. Histopathology is particularly valuable in distinguishing between contaminants, colonizers, and actual causative pathogens, as Mucorales and other opportunistic fungal pathogens are ubiquitous in the environment [31]. In some cases, histopathology may be the only evidence of fungal infection when the organism is not recovered in culture. While histopathology provides important information about tissue invasion, it is not sufficient for identifying the causative organism. Fungal pathogens often exhibit similar morphologies in tissue stains, making it challenging to differentiate them based on histopathology alone [31]. Additional methods, such as fungal culture, phenotypic and morphological analyses, and molecular testing, are necessary to accurately identify the organism. Some fungi, including *Apophysomyces* spp., may require alternative conditions to sporulate in the laboratory, posing further challenges.

Identifying the causative fungal pathogen is crucial for determining appropriate treatment strategies. Microbiology culture, serological tests, antigen detection, PCR, or sequencing may be necessary to confirm the species. Collaboration and communication between clinicians, pathologists, and microbiologists are essential for achieving a timely and accurate diagnosis, guiding appropriate treatment decisions.

Overall, this study highlights the significance of pulmonary, rhino-cerebral, gastrointestinal, and soft tissue mucormycosis as distinct clinical entities within the spectrum of mucormycosis infections. Pulmonary mucormycosis, often observed in immunocompromised individuals, represents a severe manifestation with a high mortality rate, necessitating prompt diagnosis and aggressive treatment. Rhino-cerebral mucormycosis, primarily affecting the sinuses and adjacent structures, poses a significant challenge due to its rapid progression and potential for intracranial extension. Gastrointestinal mucormycosis, though relatively rare, is associated with a unique set of clinical features and diagnostic hurdles, emphasizing the need for heightened clinical suspicion. Lastly, soft tissue mucormycosis can manifest as localized or disseminated infections, with early surgical intervention playing a pivotal role in improving outcomes. By recognizing the risk factors, distinct clinical presentations, and therapeutic considerations of these different forms of mucormycosis, clinicians can ensure prompt diagnosis and tailor their management strategies more effectively, ultimately improving patient care and outcomes in both immunocompromised and immunocompetent individuals.

## Supplementary Material

ofad527_Supplementary_Data

## Supplementary Data

Supplementary materials are available at *Open Forum Infectious Diseases* online. Consisting of data provided by the authors to benefit the reader, the posted materials are not copyedited and are the sole responsibility of the authors, so questions or comments should be addressed to the corresponding author.
