# Supplementary figures and images for: Diverse Clinical Manifestations and Challenges of Mucormycosis: Insights From Serial Cases
Source: Open Forum Infect Dis. 2023 Oct 24;10(11):ofad527. doi: 10.1093/ofid/ofad527 (PMC11320587; doi:10.1093/ofid/ofad527)

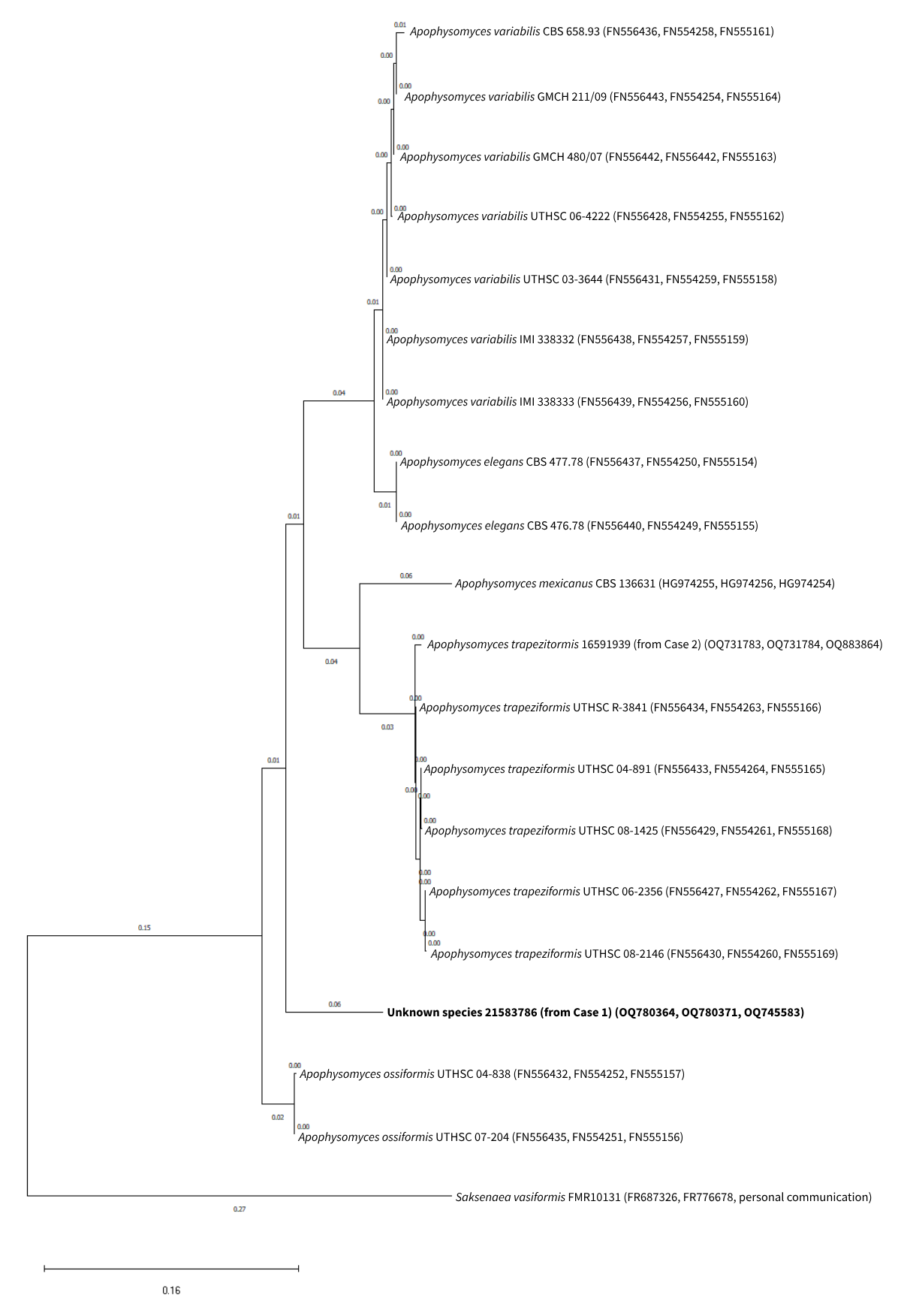

Supplement: ofad527_Supplementary_Data [file ofad527_supplementary_data.zip › OFID Supplemental Figure 3.tiff]

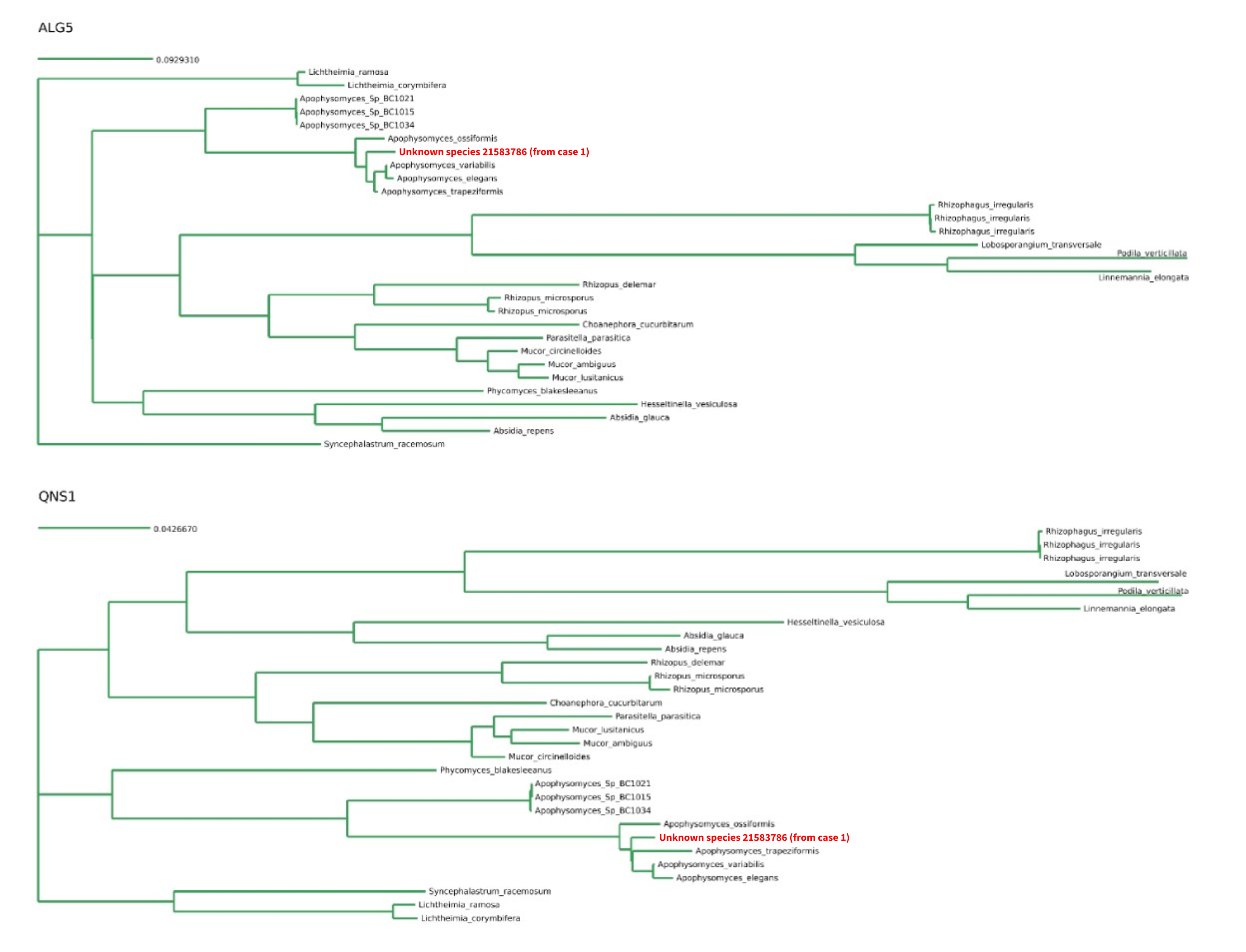

Supplement: ofad527_Supplementary_Data [file ofad527_supplementary_data.zip › OFID Supplemental Figure 4.tiff]

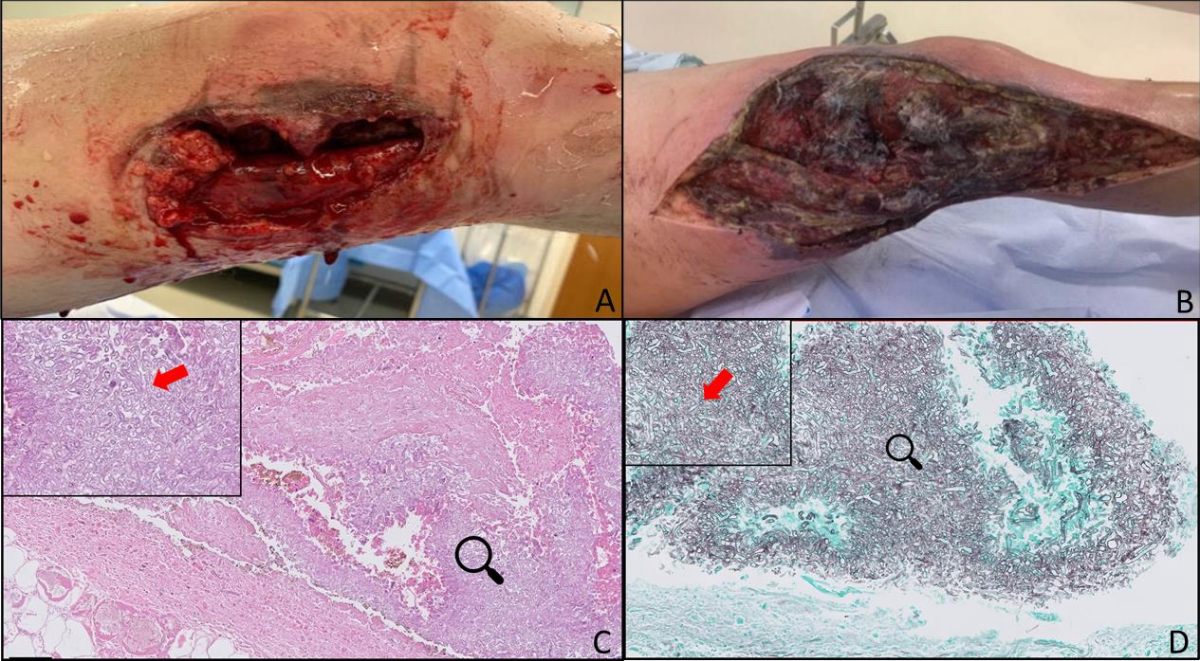

Supplement: ofad527_Supplementary_Data [file ofad527_supplementary_data.zip › OFID Supplemental Figure 1.tiff]

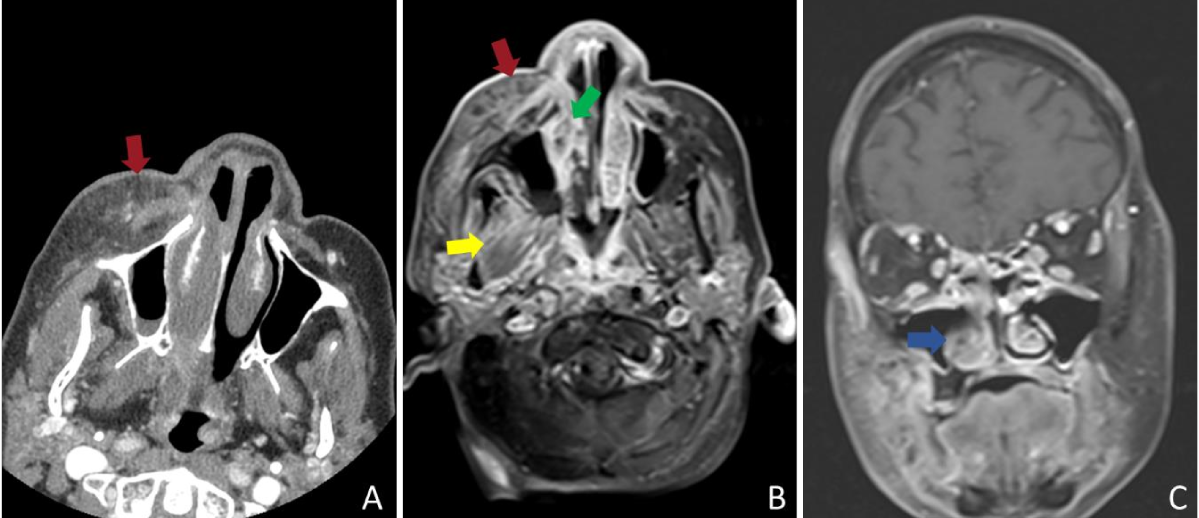

Supplement: ofad527_Supplementary_Data [file ofad527_supplementary_data.zip › OFID Supplemental Figure 2.tiff]
